# Guidance for the Interpretation of Continual Cuffless Blood Pressure Data for the Diagnosis and Management of Hypertension

**DOI:** 10.3389/fmedt.2022.899143

**Published:** 2022-05-17

**Authors:** Josep Sola, Meritxell Cortes, David Perruchoud, Bastien De Marco, Melvin D. Lobo, Cyril Pellaton, Gregoire Wuerzner, Naomi D. L. Fisher, Jay Shah

**Affiliations:** ^1^Aktiia SA, Neuchâtel, Switzerland; ^2^Barts NIHR Biomedical Research Centre, William Harvey Research Institute, Queen Mary University of London, London, United Kingdom; ^3^Division of Cardiology, Réseau Hospitalier Neuchâtelois (RHNe), Neuchâtel, Switzerland; ^4^Service of Nephrology and Hypertension, Lausanne University Hospital and University of Lausanne, Lausanne, Switzerland; ^5^Division of Endocrinology, Diabetes and Hypertension, Brigham and Women‘s Hospital, Boston, MA, United States; ^6^Division of Cardiology, Mayo Clinic Arizona, Phoenix, AZ, United States

**Keywords:** cuffless blood pressure, continuous blood pressure, home blood pressure, daytime blood pressure, hypertension, blood pressure phenotype, nocturnal blood pressure, time in target range

## Abstract

Hypertension remains the leading risk factor for death worldwide. Despite its prevalence, success of blood pressure (BP) management efforts remains elusive, and part of the difficulty lies in the tool still used to diagnose, measure, and treat hypertension: the sphygmomanometer introduced by Samuel Siegfried Karl von Basch in 1867. In recent years, there has been an explosion of devices attempting to provide estimates of BP without a cuff, overcoming many limitations of cuff-based BP monitors. Unfortunately, the differences in underlying technologies between traditional BP cuffs and newer cuffless devices, as well as hesitancy of changing a well-implemented standard, still generate understandable skepticism about and reluctance to adopt cuffless BP monitors in clinical practice. This guidance document aims to navigate the scientific and medical communities through the types of cuffless devices and present examples of robust BP data collection which are better representations of a person's true BP. It highlights the differences between data collected by cuffless and traditional cuff-based devices and provides an initial framework of interpretation of the new cuffless datasets using, as an example, a CE-marked continual cuffless BP device (Aktiia BP Monitor, Aktiia, Switzerland). Demonstration of novel BP metrics, which have the potential to change the paradigm of hypertension diagnosis and treatment, are now possible for the first time with cuffless BP monitors that provide continual readings over long periods. Widespread adoption of continual cuffless BP monitors in healthcare will require a collaborative and thoughtful process, acknowledging that the transition from a legacy to a novel medical technology will be slow. Finally, this guidance concludes with a call to action to international scientific and expert associations to include cuffless BP monitors in original scientific research and in future versions of guidelines and standards.

## Background

### The Need for a Paradigm Shift in the Control of BP

The scale of the problem of hypertension is difficult to comprehend–over 1.3 billion people worldwide are estimated to be hypertensive (1). Nevertheless, the current paradigm of diagnosis, treatment, and monitoring for hypertension (HTN) has resulted in poor rates of control (2). There is substantial risk in maintaining the status quo: HTN is and has been the single largest contributor to cardiovascular death and disease for over 40 years (3), is estimated to cost the US healthcare system $131 billion/year (4) and is the leading preventable risk factor for premature death worldwide (2–5). Any change that may improve hypertension care, given the immense potential for benefit, must be explored fully.

The past two decades have witnessed a dramatic digital shift in healthcare, enabling a more robust, real-time, and practical exchange of data and information between patients and providers, primarily through widespread adoption of the EHR and accompanied by numerous digital health applications. Indeed, 20 years ago—7 years prior to the release of the first iPhone—the Institute of Medicine (IoM) authored a book entitled *Crossing the Quality Chasm: A New Health System for the 21st Century* (6), in which the authors highlighted the potential of computer-aided (what is now termed digital health or mHealth) tools that assist in automating the transfer of clinical data to clinicians, both to improve clinical care and to further our understanding of disease (6).

Two decades later, technology is beginning to fulfill the IoM's visionary insights and call to action. Cuffless BP technology promises to improve the diagnosis, treatment, and monitoring of hypertension, carrying the potential to benefit millions of hypertensive people. Innovation has long been focused on treatment once a disease has been manifest, but now technology has afforded an opportunity to help prevent uncontrolled HTN and all its attendant risks. The commercialization of cuffless BP devices will allow us to solve many of the behavioral and practical challenges of treating a widespread chronic disease that has long languished in the background. The treatment of hypertension has been constrained by the limits of both in-office BP measurements and cuff-based home measurements. It is reasonable to expect that large scale adoption will allow realization of the benefits of cuffless BP devices, and in turn greater global hypertension control.

### Moving Beyond the Limitations of the Seated, Resting BP Methodology

In 1948, one of the most important studies of cardiovascular risk was launched in Framingham, Massachusetts. There is little doubt of the seminal nature of the insights gained from the research over the subsequent three decades. As part of the study protocol, in-office measurements of BP were determined by the only available technology at the time—auscultation of the Korotkoff sounds to estimate systolic and diastolic BP. Subjects were seated with their backs against a chair, and measurements were taken in the left arm only. To this day, except for the notable shift from the manual mercury manometer to automated oscillometric devices, all major guidelines recommend that BP measurements are taken as originally described by the Framingham Study method [with additional restrictions and warnings (7–11)].

After decades of cuff-measured targets within clinical trials and practice guidelines, this methodology of BP collection has been enshrined in collective medical thought as the standard for estimating BP. Furthermore, it has supported a hypothesis that an individual has a physiologically stable and predictable BP. Even expert consensus documents, such as the American Heart Association guidelines, state “it is generally agreed that conventional clinic readings, when made correctly, are a surrogate marker for a patient's true BP, which is conceived as the average over long periods of time, and which is thought to be the most important component of BP in determining its adverse effects (12)”.

**Figure 1:**
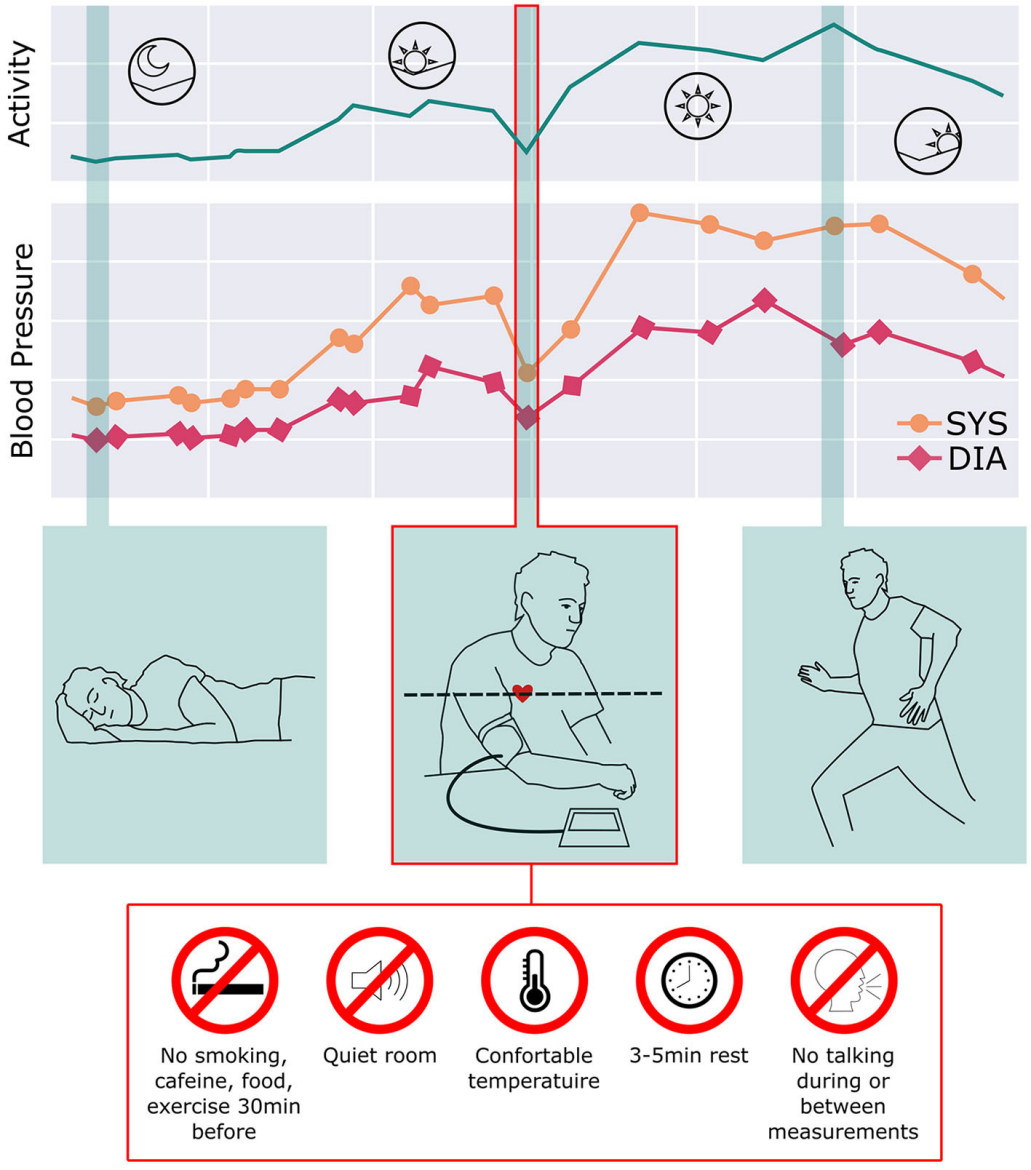
BP continuously changes and adapts over 24 h according to lifestyle, daily activities, medication treatment, and body position changes. While this reflects the natural variability of BP, current guidelines recommend obtaining BP readings using the Framingham methodology: sitting relaxed in a quiet, climate-controlled room. Given the daily variation of BP through the day, this method provides a poor representation of the BP over the day as well as long periods of time.

As the comprehension of hypertension has evolved, however, it is evident that BP continuously changes and adapts over 24 h according to lifestyle, daily activities, medication treatment, physical/emotional stressors, and body position changes. Expert guidelines also suggest that in-office readings should be confirmed by out-of-office readings over subsequent weeks and months, implying that the true nature of an individual's BP patterns in daily life cannot be wholly estimated by relaxing for 5 min in a quiet, climate-controlled environment free of exercise, speaking, caffeine, noise, and with both feet flat on the floor. Figure 1 illustrates how poorly representative a seated, relaxed BP reading is of true BP excursions over 24 h (13, 14).

Ambulatory BP monitors (ABPM) begin to show some of this variability, but usually over only 24 hours. Recently the development of continual cuffless BP devices enables, for the first time, a much more representative longitudinal depiction of an individual's BP.

### Moving Towards Systematic Measurement of BP Out of the Office

The importance of out-of-office BP measurements is recognized in all major HTN guidelines for confirming office BP readings (9, 10, 15). ABPM has been considered the gold standard for out-of-office BP measurements. However, ABPM remains woefully underutilized for a variety of reasons. While ABPM may be the current recommended tool to monitor out-of-office BP, it is almost never used in the US. The percentage of Medicare beneficiaries—for whom the expected prevalence of HTN is estimated to be 50%—with ABPM claims was only ~0.1% per year (16). In China, only 1.6% of primary care providers surveyed reported using ABPM to diagnose HTN (17). A simpler, cheaper, and more widely available solution for BP monitoring would be of significant benefit to providers and patients.

Home BP monitoring (HBPM) is also recommended by all major HTN guidelines as a critical adjunct to diagnosis, monitoring, and management for HTN (9–11). However, in practice it is difficult for patients to monitor their BP at home and send in meaningful data. In addition, patients need training to follow the same standard procedure of BP measurement as office BP measurements. The percentage of active HBPM in real-world patients is astonishingly low, despite the ready availability of relatively inexpensive home BP cuffs. Half of hypertensive patients report never checking their BP at home, 10% checked it less than once per month, and only 24% of hypertensive patients reported checking BP > 1/week (15). Data demonstrate that while HBPM is routinely recommended by expert panels and consensus guidelines, it is not actually performed by most patients with HTN, and certainly not performed twice daily for at least seven days, as recommended (9, 11).

There are many possible explanations for the marked gap between the recommendations and real-world practice. A study done in 2017 explored the barriers of primary care providers recommending HBPM to their patients. Over two thirds of respondents gave one or more of the following reasons as barriers to obtaining HBPM data (18):

a) Patients unable to complete HBPM due to low health literacy, time requirement, intrusiveness of testing, requirement of a routine, and requirement to bring HBPM to the office.
b) Test results inaccurate due to patient noncompliance with HBPM protocol (e.g. incorrect cuff size, poor timing of BP readings, failure to record readings, “cherry-picking” normal BP readings to show physicians.
c) Inaccurate results due to patient factors such as body habitus
d) Test results or cuff inflation could increase patient anxiety and hence accuracy

Continual cuffless BP devices have the potential to solve many practical and behavioral issues, to overcome barriers to recommended routine monitoring of BP, and to obtain significantly more BP data compared to traditional methods. The ability to collect continual BP readings at home, out of the office, during daily activities, and while sleeping, over periods of weeks, months, and years, gives patients and providers a far more complete assessment of BP than intermittent checks in a controlled position and environment, which provide physicians and patients only glimpses of the complete representation of BP (13, 14).

The present guidance document explores the general types of cuffless devices currently available and presents examples of data from a CE-marked continual cuffless BP monitoring device (Aktiia BP Monitor, Aktiia, Switzerland). It further explores BP metrics only possible to measure with continual cuffless devices, their potential clinical relevance, and integration into current medical systems. Given that the burden of HTN is increasing, the quality of HTN control is worsening, and that technology can now overcome many of the barriers to HTN care, now is the opportune moment for the medical community to invest in clinical research to demonstrate outcomes and for governmental agencies to act to allow innovative solutions. Finally, it provides a proposal for expert committees to incorporate cuffless BP devices and data into future guidelines and standards.

## The New Paradigm of Cuffless BP Monitors

### Definition of a Cuffless BP Monitoring Device

Before introducing the potential benefit of cuffless BP monitors in the diagnosis and management of HTN, it is important to define a cuffless BP device. The ability to obtain continual BP readings out of the office and during day and night has been traditionally limited because available monitoring technologies required the inflation of a cuff for each measurement. Devices that do not require inflation of a cuff have can overcome most of the limitations inherent in traditional BP cuff monitors.

*A cuffless BP monitoring device is defined as a device or a technology that non-invasively determines the BP of an individual without creating any arterial occlusion* (19).

**Figure 2:**
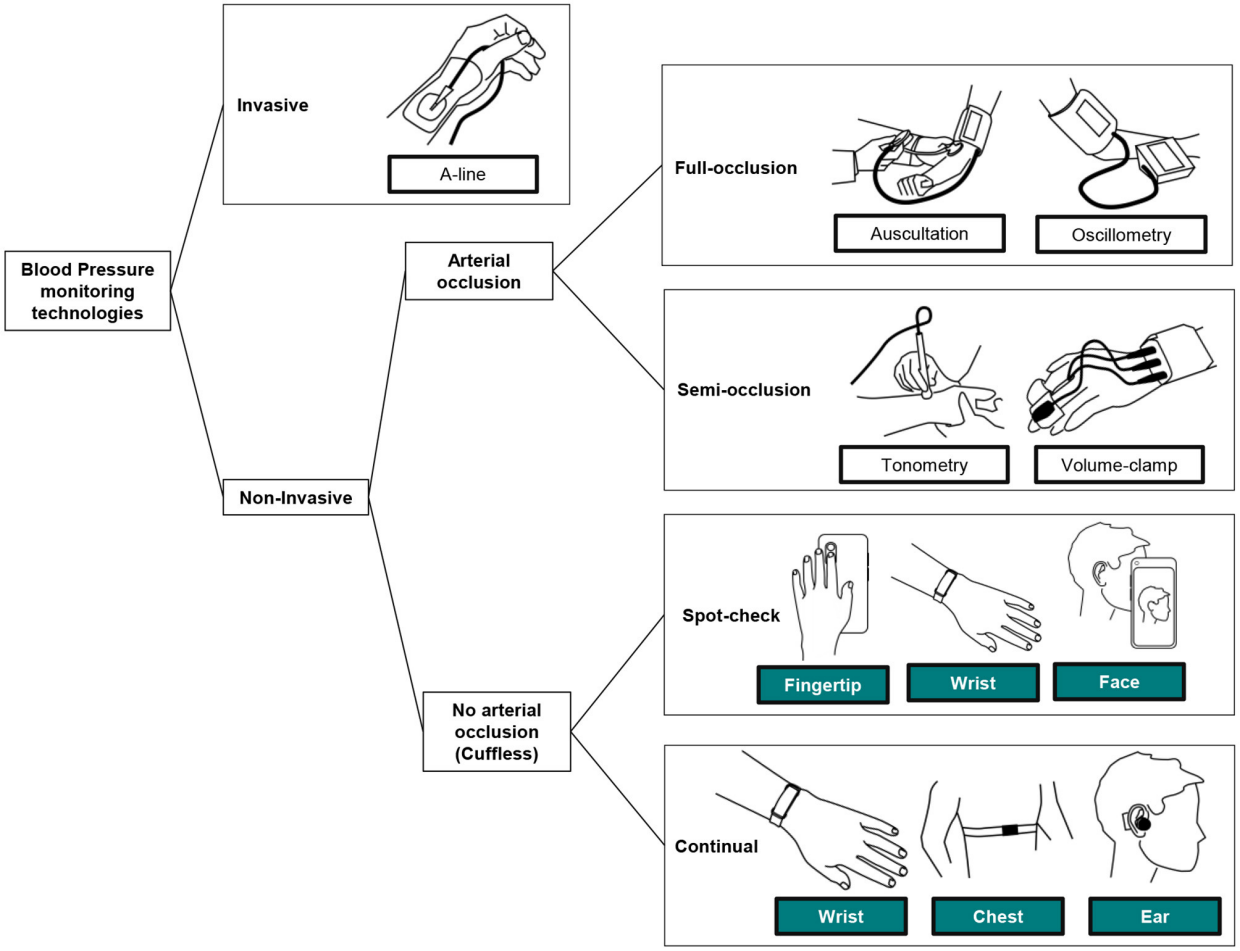
Classification of the BP monitoring technologies in clinical use today, and introduction of the perimeter of cuffless BP monitors. While spot-check monitors allow the assessment of BP readings on demand only, continual monitors can measure on demand and continuously on the background during days and nights [adapted from (19)].

To define the position of cuffless BP monitoring technologies within all BP monitoring techniques, Figure 2 provides a visual classification of the most relevant BP monitoring technologies in use today.

When patient care relies on the accurate beat-to-beat tracking of BP, invasive arterial monitoring remains the standard. Mostly used in acute medical settings such as intensive care units and operating rooms, they require insertion of a catheter into an artery, which is not safe or practical for the continual measurement of BP in the home environment.

Second, and mostly used in the diagnosis and treatment of HTN, are full-arterial occlusion techniques. Before the invention of automated sphygmomanometers, auscultation allowed large-scale BP monitoring campaigns across the globe. Still used for some in-office and in-hospital measurements, this technique requires the intervention of a practitioner who manually inflates and deflates a brachial cuff while detecting different Korotkoff sounds (arterial reverberation) with a stethoscope. Because of the technique, knowledge, and practice required to perform reliably, auscultation is not realistic for continual measurements performed at home. Since the 1970s, an alternative to auscultation gained widespread use in clinical practice: the oscillometric technique. Based on fully automated inflation and deflation of a cuff (typically placed around the upper arm), oscillometry is well-suited for home self-measurement of BP, leading to HBPM and 24 h measurement of BP (ABPM). HBPM devices require manual triggering of each reading by the patient while seated and relaxed, and ABPM devices interrupt the daily routine of the patient by regularly and frequently inflating over daytime and nighttime. The cumbersome nature of these approaches makes them unsuitable for continual BP monitoring for extended periods (20).

Semi-occlusive techniques such as tonometry (a sensor reading displacement of the skin generated by the pulsatility of an artery) and volume-clamp (a device continuously adjusting the pressure of a cuff placed around the phalanx) are used for research purposes and in some acute-care settings. Their complexity of use and very high costs precludes them from mainstream use (20).

Finally, the family of cuffless BP monitoring devices refers to those other techniques that are not invasive and do not fully or partially occlude an artery to perform each reading. Implementation of these devices largely varies (see **Table 2** for further list of available devices) and can be placed in body locations such as the wrist, fingertip, chest, ear, forehead, or a combination. Because of the different body locations, some BP monitors are practically limited to spot-check (i.e., sporadic) measurements while a few are capable of continual (day and night) measurements (19).

### What Do Cuffless BP Devices Measure Differently?

To interpret the different types of BP data generated by each BP monitoring technology, it is important to understand that each type of device measures a different physiological phenomenon. Therefore, measurements simultaneously acquired on the same patient by different technologies may be different (Table 1 provides an overview of these differences).

**Table 1:** Measurement principles and measured quantities by different BP monitoring technologies [definitions adapted from (19)].

**BP monitoring family**	**Measurement technology**	**Measured quantity**	**Matching to pressure (in mmHg)**	**Type of measurement**
Invasive	Pressure sensor in the catheter (measurements expressed in mmHg)	Local arterial pressure	Direct	Measurement
Auscultation	Ear: acoustic wave heard by an observer (manual acoustic observations)	Time of onset of the acoustic wave on a stethoscope	Visual inspection of the pressure of a manually deflated sphygmomanometer	Estimation
Oscillometry	Pressure sensor measuring the pneumatic pressure within a cuff (measurements expressed in mmHg)	Time of onset of a characteristic point in the amplitude of pulse-related volume changes of a limb	Automatic matching to the actual pressure of a sphygmomanometer at the onset time	Estimation
Cuffless device	Pressure-less sensor measuring the waveform of the pulsatility of an artery or arterial bed (measurements expressed in mV from an optical, biopotential, or radar sensor)	Quantity from the analysis of the waveform of the pulsatility of an artery or arterial bed	Initialization process involving an external BP measurement (typically from an oscillometric device)	Estimation

The only technique that directly measures the BP in mmHg (or kPa) is arterial catheterization. Invasive techniques provide a genuine pressure reading of the actual fluid pressure within the artery at the location of the catheter. However, even invasive arterial measurements can vary: two simultaneous invasive readings from two different arteries (e.g., the radial artery and the ascending aorta) can provide largely different BP values because of arterial amplification and hydrostatic pressure phenomena (21).

Although auscultatory and oscillometric readings of BP tend to be discussed interchangeably in clinical practice, these techniques measure two different phenomena, neither of which is a direct pressure. Auscultation provides a complex multi-modal estimation of BP that relies on the genesis of acoustic waves created by arterial reverberation when it is released from a full occlusion. The actual estimation of BP by auscultation is based on the audible fluid wave that an observer matches to the instantaneous pressure of a sphygmomanometer that is being manually deflated (20).

Oscillometry provides a pneumatic estimation of BP that relies on the identification of patterns on the changes of volume of the arm (or wrist) while the inner arteries are exposed to different intramural pressures. An oscillometric device thus does not directly measure a pressure, but a characteristic point that a computer program identifies in the amplitude of the pulse-related volume changes of the arm and that is further matched to the pressure of a sphygmomanometer being deflated (or inflated) by an electronic controller to generate a numerical BP value (20).

Cuffless BP monitors provide an indirect estimation of BP that relies on the analysis of the arterial pulses at one or more body location(s) with a sensor that applies no pressure to that location. Cuffless BP monitors provide not a direct pressure measurement, but a quantity that a computer program calculates from the analysis of the waveform of a pressure pulse which is mapped to a BP value typically following an initialization phase (19). Several sensor technologies are currently used to capture the waveforms of pressure pulses ranging from optical sensors [assessing the pulsatility of skin arterioles via reflection or transmission photo-plethysmographic sensors (22)], camera sensors [assessing the pulsatility of skin arterioles via reflection video-based photo-plethysmography (23)], biopotential sensors [assessing different electro-magnetic signatures of the cardiac activity, or assessing arterial pulsatility from impedance plethysmography signals at different body locations (24)], radar sensors [assessing arterial pulsatility at different body locations from radar reflections (25)] and tonometric sensors [assessing pulsatility of superficial arteries by sensing displacements of the skin (26)]. Depending on the number of body locations on which a pressure pulse is captured on the patient body, the analysis of the waveforms is performed based either on pulse wave velocity algorithms (typically when at least two body locations are involved) or on pulse wave analysis algorithms (typically when one single body location is involved). Because no pressure measurement is involved in the assessment of such pulsatility waveforms, most cuffless BP monitor still require an initialization procedure that involves the use of an oscillometric device to provide information in “mmHg”.

### What Cuffless BP Devices Are Available Today?

At the time of writing, the following cuffless BP monitors are available on the market. Table 2 provides a classification of those devices based on different criteria previously discussed in this section.

**Table 2:** List of current commercially available cuffless BP monitoring devices.

**Cuffless BP monitor**	**Intended use**	**Time of use**	**Cuff calibration/cuff included**	**Episodic/continual measurement**	**Location of sensor**
ViSi Mobile System Sotera Wireless, US	Acute care (hospital)	Short-term	Yes/No	Continual	Chest and thumb
Aktiia 24/7 BP Monitor Aktiia, Switzerland	Home use	Long-term	Yes/Yes	Continual	Wrist
Galaxy Watch Samsung, Korea	Home use	Long-term	Yes/No	Episodic	Wrist
Somnotouch NIBP Somnomedics, Germany	24-h ambulatory BP monitor	Short-term	Yes/Yes	Episodic (24 h period)	Chest/wrist
Biobeat Chest/Wrist Monitor Biobeat, Israel	24-h ambulatory BP monitor	Short-term	Yes/No	Episodic (24-h period)	Chest/wrist
Caretaker 4 Caretaker, US	Acute care (hospital)	Short-term	No/No	Continual	Wrist/finger
BPro G2 Healthstats, Singapore	24-h ambulatory BP	Short-term	Yes/No	Episodic (24 h period)	Wrist (radial artery)

### The Validation and Reliability of Cuffless BP Monitors

While the trust of the accuracy of BP readings provided by automated oscillometric cuffs is supported by the existence of well-defined and recognized international standards for the design and performance of validation clinical trials (27, 28), there is to date no recognized and harmonized standard that specifies how a cuffless BP monitor should be clinically validated. It is important to highlight that validation protocols specifically designed to assess oscillometric devices are inappropriate for the validation of cuffless BP monitors (29).

Because different cuffless BP monitors rely on different technologies, some of which require calibration procedures before being used, and their intended uses may range from ambulatory (home) monitoring to acute-care (hospital) monitoring, the design of a universal and recognized validation framework across devices has not yet been achieved. Nevertheless, and over the last decade, different initiatives have emerged to provide guidance on how to clinically validate some of these devices and technologies. With no intention to extensively cover all published materials, we enumerate here the most relevant initiatives:

- In 2014 Institute of Electrical and Electronics Engineers (IEEE) issued a protocol for the validation of wearable cuffless BP monitors that was further amended in 2019, entitled “IEEE 1708 IEEE Standard for Wearable Cuffless Blood Pressure Measuring Devices” (30)
- In 2021 International Organization for Standardization (ISO) issued a draft version of a protocol for the validation of continuous automated BP monitors, entitled “ISO 81060-3 Non-invasive sphygmomanometers—Part 3: Clinical investigation of continuous automated measurement type” (31)
- In 2021 Mukkamala et al. issued a joint statement on the challenges and proposals for the evaluation of the accuracy of cuffless BP measurement devices (9, 29)

As there is not yet an international standard that covers the validation of cuffless BP monitors, they are commonly subject to questions of reliability of their readings. Fortunately, in contrast with wearable and fitness devices (heart rate monitors, activity trackers and sleep trackers), BP monitors fall into the category of medical devices—legally marketed devices which require the approval of country-specific regulatory bodies before they are available to patients and providers. The approval process varies across countries (32, 33) but in general requires demonstration of a favorable benefit-risk determination of the device (34). For example, while a very accurate cuffless device that exposes the user to radiation would not be acceptable for long-term monitoring, the same device might have greater benefit than risk when used in short-term monitoring of severe and acute conditions. Similarly, a slightly less accurate cuffless device that remained unobtrusive and easy to use might pass acceptable thresholds when used in long-term monitoring of BP trends in real-world settings. In this instance, the requirements of single-measurement accuracy of traditional cuffs may be traded for the ease of use of a cuffless device and the improved patient compliance when used in monitoring HTN over the long-term.

In summary, while the lack of accuracy validation standards might be initially seen as a barrier for the adoption of cuffless BP monitors in clinical practice, their ability to generate longitudinal metrics of BP control for patients in an ambulatory setting has the potential to shift the field of hypertension management to focus less on highly-accurate but episodic readings and more on the continual and longitudinal BP data. Nevertheless, future clinical outcomes research will be required to establish new guidelines and treatment strategies for the optimal use of these devices in real-life interventions.

### Standard Cuff Measurements Provide Only Glimpses of the State of Disease

A major appealing feature of continual cuffless BP devices is the potential to provide significantly more BP data points. The ability to collect continual BP readings at home, out of the office, in daily life, and at night and while sleeping, over periods of days to years, gives patients and providers a far more representative assessment of BP than occasional cuff estimates (13, 14). The snapshots of BP measured in-office or by HBPM at one point in time represent only a fraction of the full dynamic data set of BPs. Without these data, physicians and patients are essentially blind to the true nature of BP.

**Figure 3:**
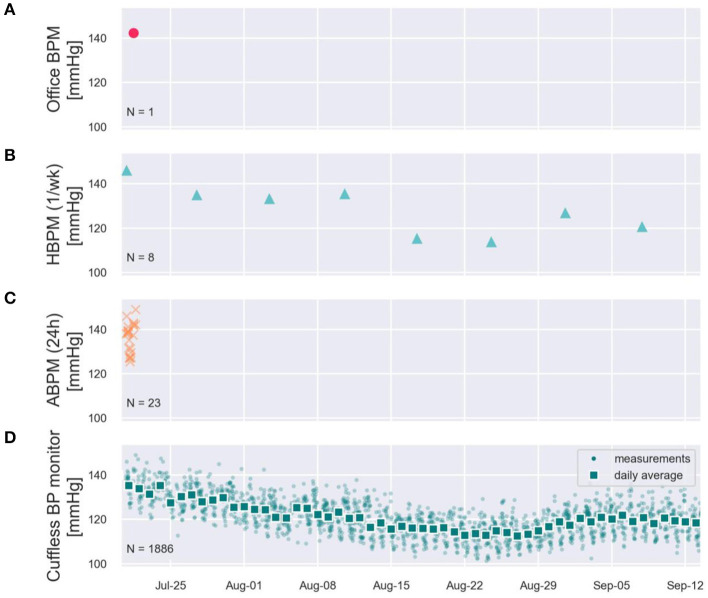
Simulated office **(A)**, Simulated HBPM once/week **(B)**, simulated ABPM **(C)**, and two months of measured continual cuffless BP data **(D)** on a single user, depicting typical day-night excursions of Systolic BP of 10 mmHg (night dip), and a superposed decreasing trend of BP across the summer weeks.

To illustrate these limitations, Figure 3 provides real-world systolic BP (SBP) data recorded on a male subject using a CE-marked continual cuffless BP device over 2 months. The data demonstrate the stark disparity between simulated in-office readings, occasional home BP monitoring, and ambulatory BP monitoring. The in-office estimate suggests a significantly higher absolute SBP value than the average and does not capture longitudinal BP data, demonstrated most clearly and commonly in white-coat and masked HTN syndromes (up to 40% of individuals). Home BP estimates of SBP—when performed routinely—may correlate with overall averages, but do not capture the daily and circadian variability. Finally, an ambulatory BP monitor reveals information during only a narrow (24- or 48-h) period. All three traditional methods of BP estimates represent only glimpses into the dynamic BP, which is demonstrated very well by the continual cuffless device.

## A Guide to the Interpretation of Continual Cuffless BP Data

Current practicing clinicians have spent years in medical school and post-graduate training, reading guidelines and clinical trials, and treating their patients in practice with a framework of the traditional sphygmomanometer to measure, record, diagnose, and treat BP. The BP cuff is a cornerstone of performing the first step of any basic physical exam, appropriately labeled, “the vital signs.” To alter the method of so foundational an aspect of medical care requires providers to have a complete understanding of how data from newer technology differs, and therefore, how to incorporate the data into their overall clinical assessment. Useful novel medical technology should also provide better data; therefore, this guide aims to present a framework which a clinician may apply to validated cuffless BP monitoring devices, particularly focusing on the interpretation of continual cuffless BP data.

### Interpretation in the Context of Legacy Modalities

HBPM and ABPM remain the recommended methods of measuring out-of-office BP in clinical practice and their data are often the primary basis of changes in BP management. It is also recognized that out-of-office measurements are not always consistent with office measurements, and the precise relationship between modalities and settings remains “unsettled.” With this rationale, the 2017 ACC/AHA HTN guidelines give a specific guidance statement on how to compare OBP, HBPM, and ABPM readings, going so far as to specify different threshold values for the stages of BP depending on the device and setting, and even the definition of stage 1 HTN differs between guidelines (11, 15). Applying the same logic to the new class of cuffless BP monitors, similar guidance is necessary to place cuffless data into the context of established BP techniques and thresholds.

#### Comparing HBPM and Cuffless BP Monitors

HBPM is currently the most prescribed method to obtain out-of-office BP readings. Although guidelines on how and when BP readings should be performed vary across countries and guidelines, the following recommendations are common in all guidelines (9, 10): a patient uses a validated HBPM device to perform a BP measurement at least once every morning (or twice daily) after relaxing quietly for 5 min in the sitting position. The measurement consists of taking at least 2 consecutive BP readings and averaging them, the patient records the BP values in some way (diary, App, or spreadsheet), and finally the patient needs to remember to bring the diary, App, or the BP monitor itself (and its stored memory of readings) to the office for their next provider visit.

**Figure 4:**
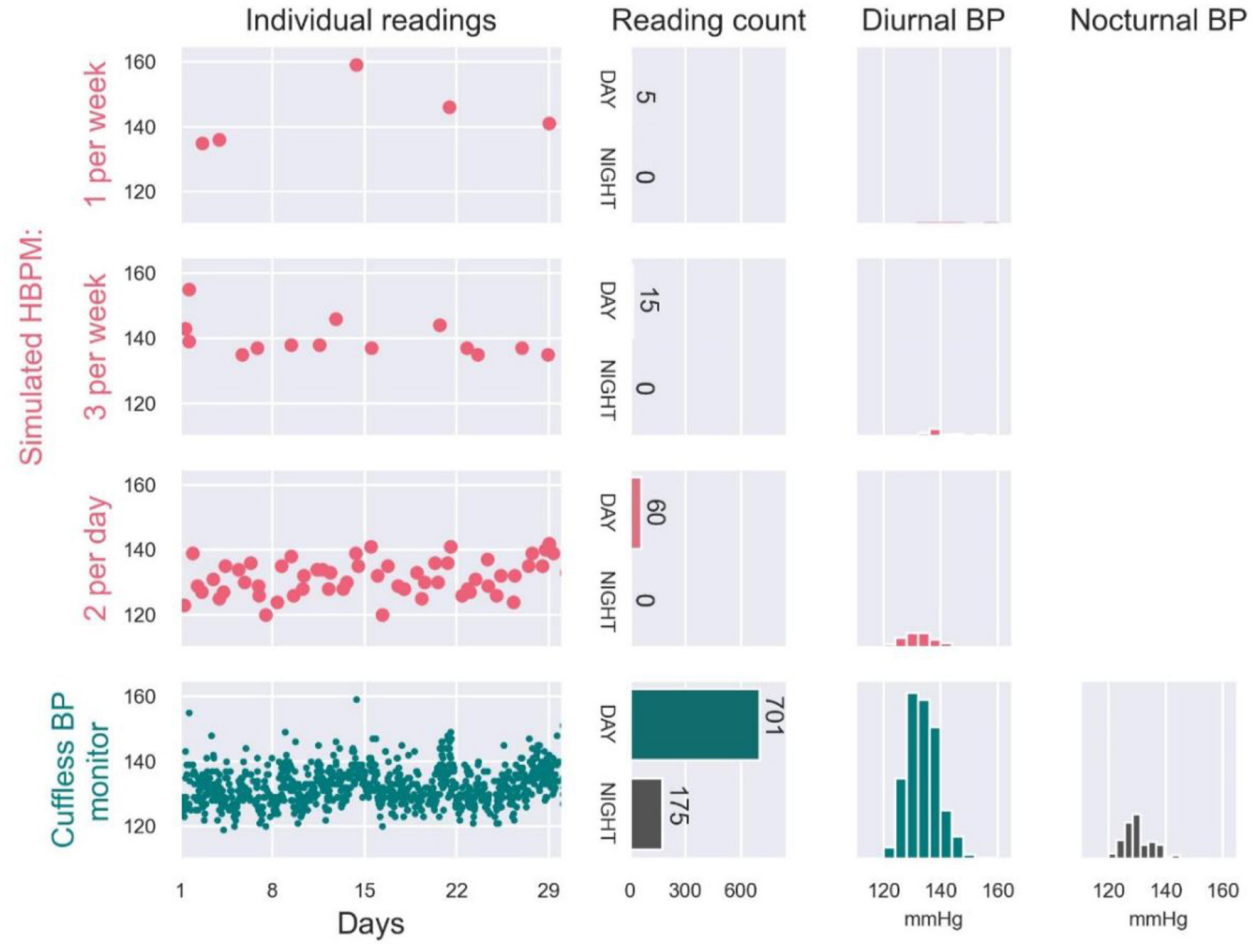
Real-world SBP data collected on a patient by a continual cuffless BP monitor over one month (bottom panel, green), and simulated examples of HBPM readings that would have been recorded by the patient when performing 1 measurement per week (first panel, pink), 3 measurements per week (second panel, pink) and 2 measurements per day (third panel, pink). Simulated examples were generated by randomly resampling the measured continual BP data.

Even a motivated patient following a guideline-based measurement routine with HBPM usually does not provide more than 30 BP daytime readings/month, and cannot practically measure nocturnal BP (16). The intermittent nature of these readings makes HBPM generally unable to capture the variability of BP. A simulated example of this limitations of HBPM is provided in Figure 4. From a user wearing a continual cuffless BP device, 974 readings acquired over 1 month were used as a benchmark of real-world BP variability. The full dataset allowed accurate estimation of daytime (*N* = 701 readings) and nighttime (*N* = 175 readings) averages of SBP (133 mmHg and 131 mmHg respectively). From the same readings, we simulated the data that the patient would have brought to the provider from an HBPM device: the simulated HBPM measurements were constructed by randomly resampling the continual cuffless data following three typical HBPM measurement schedules (data selected only during daytime and at different periodicities). According to the illustrated simulation, the daytime averages of SBP would have been of 143 mmHg (measured 1/week), 140 mmHg (3/week) and 133 mmHg (2/day) respectively. In addition to the inaccuracy of the reported daytime averages, the HBPM-simulated readings would have also missed significant BP excursions (Day 21 and 22), unless the patient had been using the HBPM monitor daily.

**Figure 5:**
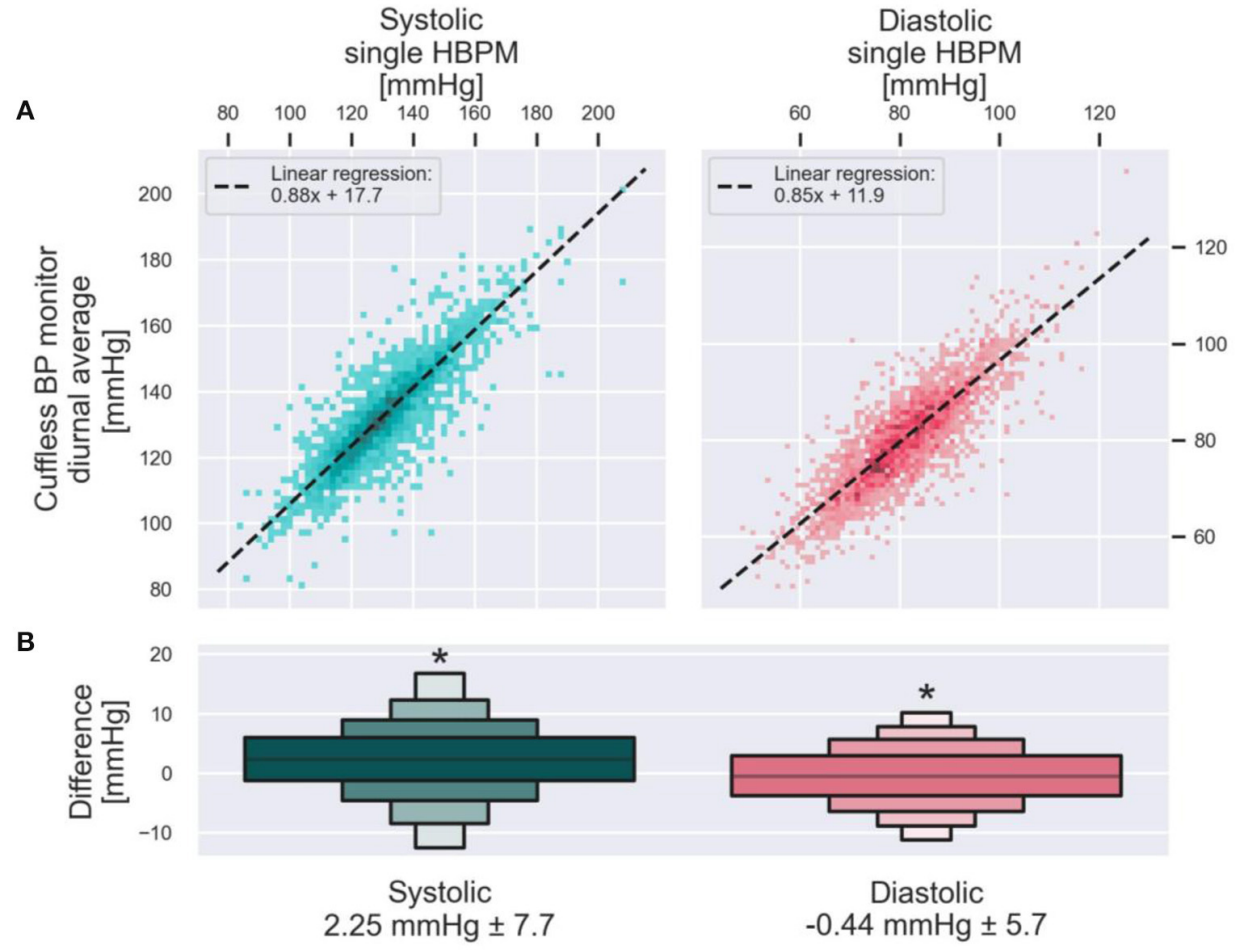
Population comparison of daytime BP averages by a cuffless BP device against the HBPM measurements obtained during the initialization of the device on the same day. **(A)** Presents one data point from each of the 2,928 users of a continual cuffless BP monitor. On the X axis is the single measure from an HBPM read during the initialization of the device, compared on the Y axis with the concurrent diurnal (8am−8pm) average BP measured by the cuffless device. Left, SBP. Right, DBP. The dotted line is calculated with a Huber linear regression. The letter-value plot on **(B)** depicts the distribution of the pairwise differences between the cuffless BP monitor values and the corresponding HBPM values. Mean and standard deviation of each distribution are presented on the bottom. Asterisks denotes statistically significant differences between the HBPM and cuffless BP monitors measurements (both *p* < 0.001).

Cuffless BP monitors can thus provide markedly more BP readings, demonstrate better the variability of BP, and provide nighttime BP measures, all of which have meaningful clinical implications. A clinician may wonder, however, how the daytime average BP provided by a continual cuffless BP monitor compares to an HBPM reading performed on the same day? To answer this question, the anonymized data from 2,928 users of a continual cuffless BP device was analyzed offline (Figure 5). The analysis compared diurnal BP data (between 8 a.m. and 8 p.m.) measured by the cuffless BP device on the day of the initialization procedure, against the brachial cuff BP measurement (HBPM) obtained during same initialization procedure. The analysis was repeated both for systolic and diastolic BP. In both cases, a paired *t*-test showed that the difference in measurements with the two modalities was statistically significant (both *p* < 0.001), although the differences (SBP of 2.25 mmHg and DBP of 0.44 mmHg) were below the resolution and error margin of any automated oscillometric BP monitor.

Using real-world data from over 2,000 patients using a commercially available continual cuffless monitor, the distinct advantages of a markedly richer BP data set, the ability to measure nocturnal BP longitudinally, and the automated, passive nature of the device are striking when compared to traditional monitoring (Table 3). Furthermore, the systematic differences of daytime BP averages of the continual cuffless monitor as compared to daytime HBPM readings across this population are small and within an acceptable margin of error. In summary, this type of continual, cuffless device has tremendous potential to greatly improve the ability to monitor BP in the ambulatory setting.

**Table 3:** Guidance for the interpretation of daytime averages obtained from a continual cuffless BP monitor when compared to daytime averages obtained from HBPM.

**Criterion**	**HBPM**	**Continual cuffless BP monitor**
Triggering of readings	Manually triggered by the patient	Automated, imperceptible by the patient
Frequency of readings	As low as once a month to twice daily (highly compliant patient)	On average, approximately one reading per hour (avg from 4,887 users of the continual cuffless BP monitor)
Conditions of measurement during daytime	Patient is sitting and relaxed with the arm at the heart level	Anytime the patient is quiet or performs no important movement (motion tolerance might vary across devices) with no control of body and arm position
Availability of nighttime readings	No	Yes
Feasibility of long-term monitoring	Yes, but only for patients with high compliance	Yes, even for patients with reduced compliance
Systematic difference of daytime BP averages	Daytime SBP average is similar (2.2 mmHg higher) to same-day HBPM;	
(Cuffless vs. HBPM)	Daytime DBP average is similar (0.44 mmHg lower) to same-day HBPM	

#### Comparing ABPM and Cuffless BP Monitors

ABPM remains the recommended modality when more complete analysis of a patient's BP pattern is required for the diagnosis/monitoring of HTN, despite its low utilization in clinical practice (see previous sections). Figure 3 visually demonstrates the fundamental differences in the flow of data that OBP, HBPM, ABPM, and Cuffless BP monitors can generate on a given patient. While ABPM is the only currently recommended modality able to obtain day and night BP readings, its infrequent use raises questions of reproducibility. Circadian excursions of BP are known to be dynamic, and by arbitrarily picking a short monitoring period (24 or 48 h), a clinician may obtain data representative of only a narrow sliver of the overall BP (35).

**Figure 6:**
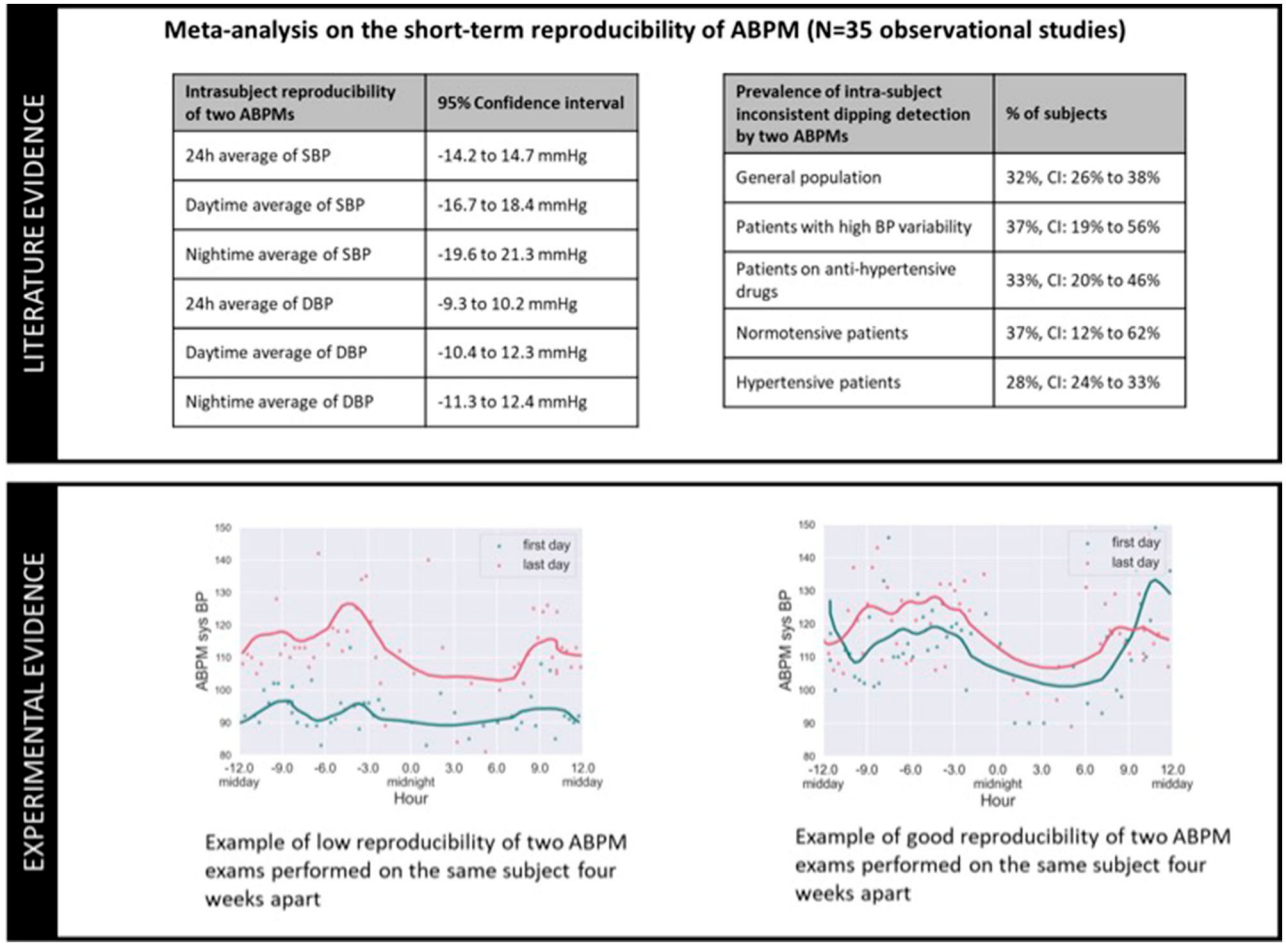
Illustration of the poor intra-subject reproducibility of ABPM exams: because the circadian BP excursions of an individual varies over time, by choosing arbitrary day(s) of measurement, an ABPM exam might generate a phenotype that misrepresents the underlying BP phenotype of the patient.

To further illustrate the reproducibility problem of ABPM, Figure 6 includes data from a meta-analysis (36) on the intra-subject reproducibility of ABPMs (upper panel), and two examples of repeated ABPM recordings from a running clinical trial (lower panel) (37). The meta-analysis of 35 observational studies demonstrates that for 1/3 of the patients, the classification of dipper/non-dipper status is not reproducible for two consecutive ABPM nights (e.g., on the first night a patient is classified as a dipper, but on the following night is classified as a non-dipper), and that the observed differences of nocturnal averages of SBP and DBP between two consecutive nights can vary between −19.6 and 21.3 mmHg, and −11.3 and 12.3 mmHg respectively. These data confirm the reproducibility of ABPM on assessing intra-individual dipping status and daytime and nighttime BP values is limited. The lower panel of Figure 6 provides ABPM recordings from selected patients of an ABPM study, the graph on left presenting very poor intra-subject reproducibility, and the graph on right presenting better reproducibility on the measured night dip and daytime/nighttime averages of BP.

Given the documented poor reproducibility of ABPMs, continual cuffless BP monitors may overcome the arbitrary nature of cuff-based ABPMs by exploiting the ability of cuffless BP to generate voluminous data over the long-term. However, comparison of data between the two modalities requires consideration of two factors. First, cuffless BP monitors fundamentally measure BP differently than those measured by traditional oscillometric ABPMs (see Table 1). Second, frequency and period of ABPM measurements (e.g., once every 20 min) differs from that of most continual cuffless BP monitors (e.g., when the user is still for long enough). This difference captures BP during different daily activities, with ABPM largely capturing more readings during physically active periods than cuffless BP devices. These two differences (technological and triggering timing) are thus expected to generate daytime and nighttime averages of BP that might differ between modalities.

**Figure 7:**
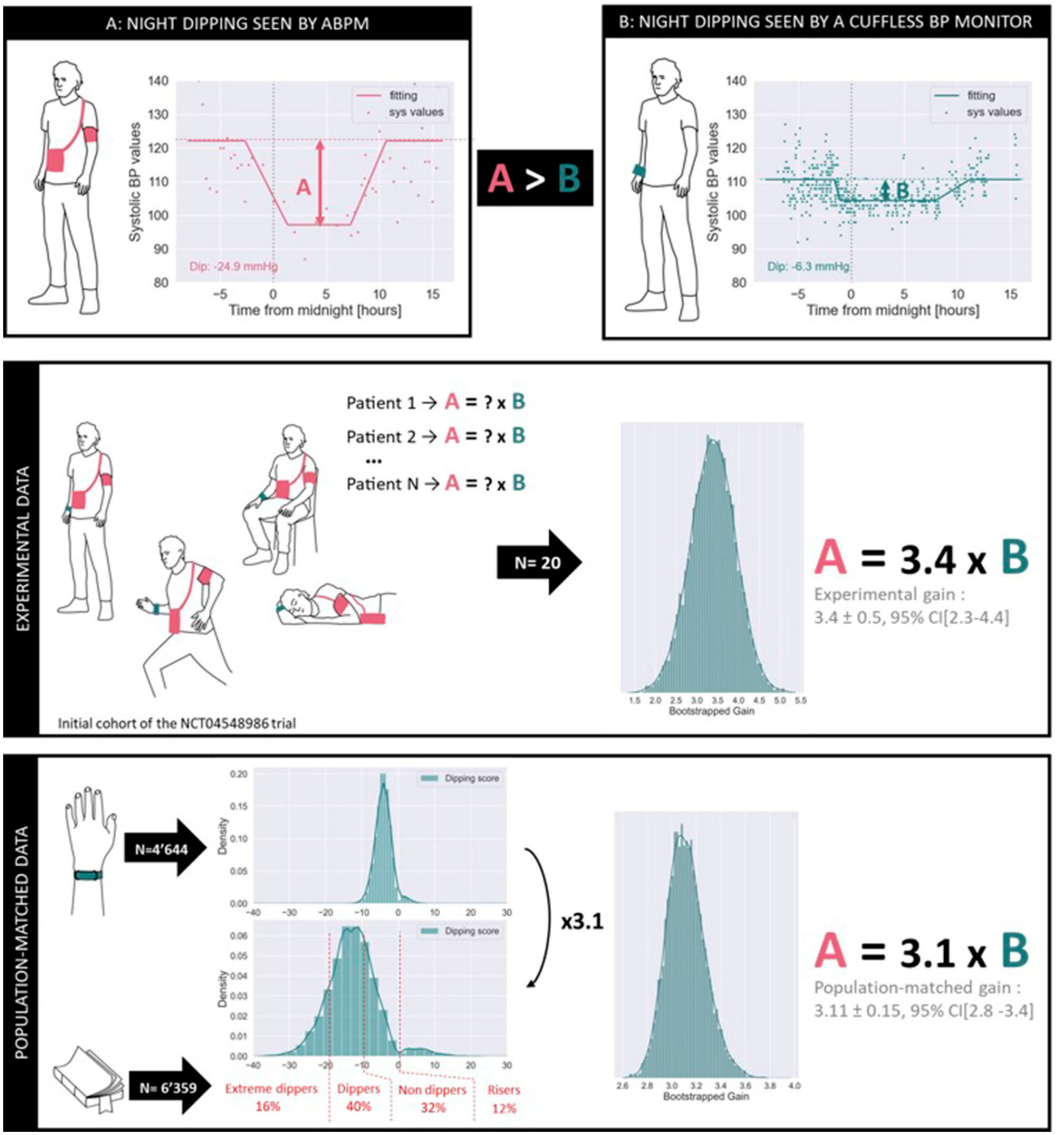
Comparison of the estimation of nocturnal dipping as measured by ABPM and by a continual cuffless BP monitor. The upper panel illustrates differences in estimated SBP dips on a patient of the NCT04548986 trial. The middle panel demonstrates a systematic factor of 3.4 across the initial cohort of patients of the same trial. The lower panel demonstrates a systematic factor of 3.1 across *N* = 4,644 users of the same commercial cuffless BP monitor when matching the phenotype distributions to that of a large independent trial (*N* = 6,359). As expected, different monitoring modalities tend to provide similar phenotyping information but require the application of technology-dependent conversion factors.

Figure 7 provides a first glimpse on the systematic differences observed between ABPM and cuffless BP monitors when estimating night dipping status of patients. The upper panel illustrates an example of simultaneous BP data from one patient enrolled in the NCT04548986 trial acquired by an ABPM monitor (Diasys 3 Plus, Novacor, France) and a continual cuffless BP monitor (Aktiia BP Monitor, Aktiia, Switzerland). Note that while the ABPM data was recorded over 24 h, the continual cuffless BP data was recorded over 1 week during and following the ABPM recordings. The monitoring period with the cuffless BP monitor was extended to 1 week to account for the day-to-day variability of circadian patterns, and to increase the number of data points registered during daytime and nighttime (because of the lower sampling frequency of the continual cuffless monitor). On the same plot, two estimates of BP dipping are extracted. To calculate the dipping on the ABPM records a common approach of the difference between daytime and night-time BP was used (38). Daytime and night-time subperiods were defined based on fixed clock-time intervals: 9 a.m. to 9 p.m. for daytime and 12 a.m. to 6 a.m. for nighttime. Data recorded during the transitional periods were excluded to avoid too much dispersion between individual users. Data points placed at higher distances than the interquartile range from the median value of each subperiod were considered as outliers, and finally, the dip was calculated as the difference between the subperiod medians. To calculate the dipping registered by the cuffless BP monitor, a statistical approach was implemented. Exploiting the fact that the cuffless circadian plot presents a higher density of data points, a parametric model was used to fit the circadian rhythm for SBP and DBP (see continuous “fitting” line in the plot): the night dip was then extracted from one of the model parameters. The estimated night dip for this patient already differs between the two modalities, the ABPM dip (A) appearing to be larger than the Cuffless dip (B).

The intermediate panel of Figure 7 provides a statistical analysis of the A > B phenomenon on a preliminary cohort of patients of the NCT04548986 trial. The data from the initial *N* = 20 enrolled patients were processed to estimate the systematic gain difference in the dipping measured by ABPM compared to the dipping measured by the continual cuffless BP monitor. In this cohort, and after bootstrapping the available samples, we calculated that the ABPM dip is characteristically 3.4 times bigger than the cuffless dip, with a 95% confidence interval ranging from 2.3 to 4.4. It is important to note that the NCT04548986 is not completed, and that a comprehensive analysis of the collected data will be further presented in a dedicated publication.

The lower panel of Figure 7 provides a further statistical investigation of the same A > B phenomenon, now merging data from an independent ABPM study on *N* = 6,359 patients and real-world data from *N* = 4,644 users of a commercial continual cuffless BP monitor. According to Kario et al., in a general population one would expect to observe the following distribution of night dipping phenotypes as measured by ABPM measurements (a phenotype is defined as an observable characteristic in the circadian patterns of BP): 16% of individuals are extreme dippers (dipping larger than 20 mmHg), 40% of individuals are normal dippers (dipping between 10 and 20 mmHg), 32% of individuals are non-dippers (dipping between 0 and 10 mmHg) and 12% of individuals are risers (positive dipping) (39). However, when observing the dipping patterns recorded by the cuffless BP monitor, the same distributions are not met, with a clear compression of the dipping distribution. The present analysis consisted of estimating the optimal factor required to expand the dipping distribution of the continual cuffless BP monitor to an ABPM-equivalent representation of phenotypes. In this cohort of *N* = 4,644 users, and after bootstrapping, we calculated that the ABPM dip is characteristically 3.1 bigger than the continual cuffless dip, with a 95% confidence interval ranging from 2.8 to 3.4.

By merging real-world data from over 4,000 users of a commercially available continual cuffless monitor with clinical data from a controlled clinical trial, it is demonstrated that continual cuffless BP monitors can estimate patients' BP phenotype. However, because of technological differences between the cuffless technology and the oscillometric ABPM monitors, technology-dependent conversion factors might be needed to compare estimates from both modalities. Table 4 provides a summarized guidance on how to interpret BP phenotyping data from a cuffless BP monitor when compared to BP characteristics obtained from ABPMs.

**Table 4:** Guidance for the interpretation of BP phenotyping data obtained from a continual cuffless BP monitor when compared to BP characteristics obtained from ABPM.

**Criterion**	**ABPM**	**Cuffless BP monitor**
Triggering of readings	Automated, perceived by the patient	Automated, imperceptible by the patient
Frequency of readings	Every 30 minutes during daytime, every 1 h during nighttime (might vary across devices and guidelines)	On average, approximately one reading per hour
Conditions of measurement during daytime	Anytime a measurement is triggered and the patient is not performing important movements	Anytime the patient is quiet or performs no important movement (motion tolerance might vary across devices) with no control of body and arm position
Availability of nighttime readings	Yes	Yes
Feasibility of long-term monitoring	No	Yes, even for patients with reduced compliance
Systematic difference of calculated night dips	SBP night dips of ABPM are ~3.2x larger than those of the Cuffless BP monitor, with 95% confidence interval ranging from 2.3 to 4.4; DBP night dips of ABPM are ~2.8x larger than those of the Cuffless BP monitor, with 95% confidence interval ranging from 1.9 to 3.8	

### Introducing a New Generation of Metrics of BP Control Based on Data From Cuffless BP Monitors

#### Dynamic Metrics of BP Control

Every time a novel monitoring modality is introduced in clinical practice, the first and obvious reaction of medical stakeholders is to compare the information provided by the new modality against the information available by the standards of care. When HBPM was first introduced in 1984, hypertension experts attempted to match the out-of-office oscillometric readings against the auscultatory readings performed during medical visits (40). When ABPM fully automated devices were further introduced, ABPM records were compared to HBPM and office readings. But every new modality further contributed to setting a new milestone consisting of either novel patient/provider interfaces or the introduction of a new generation of BP metrics. The introduction of continual cuffless BP monitoring in clinical practice will have the same effect. Hypertension research, guidelines and clinical practice have been dominated for decades by the tradition of employing one or two BP readings taken at a single point in time, mostly in the office, to represent BP control. These limited data collections were the necessary consequence of limitations with available technology to measure BP. Yet experts understand that BP is far from static. One of the biggest benefits of continual cuffless devices is their ability to provide an objective assessment of the true dynamic nature of BP. Novel concepts such as BP time in target range (TTR), BP variability, nocturnal hypertension, and BP phenotype now can be measured and targeted.

**Figure 8:**
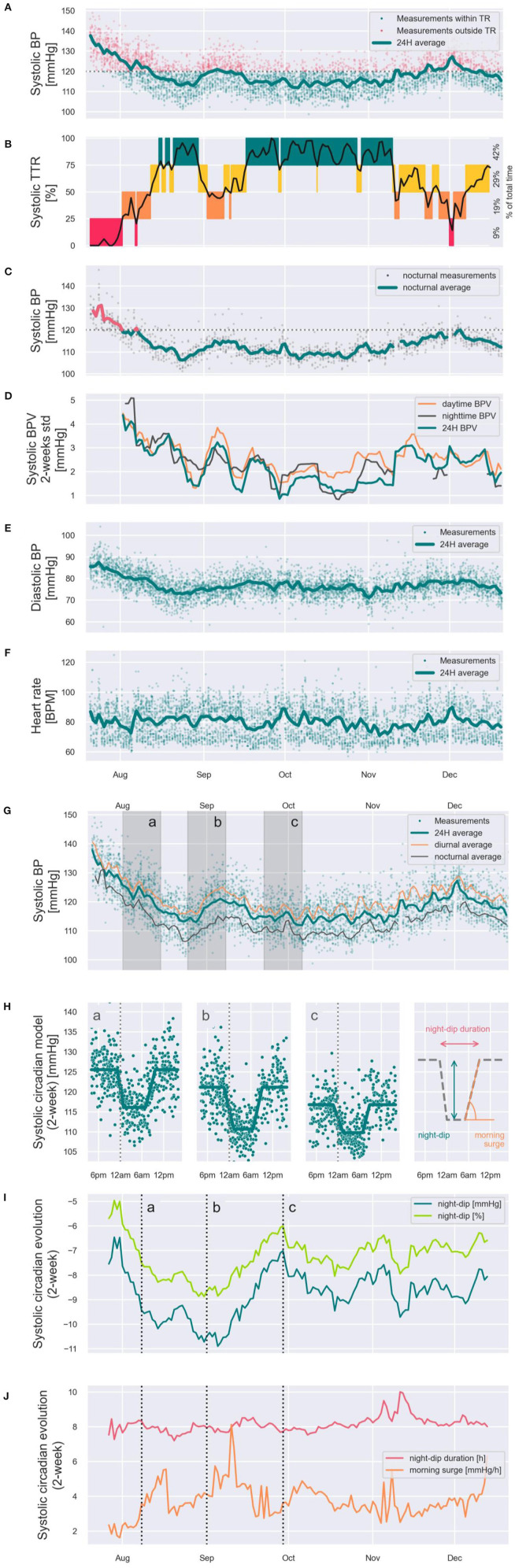
Showcasing of the new generation of dynamic BP control metrics that can be generated from the data captured from continual cuffless BP monitors. The presented time series were captured on a subject over 5 months, and in addition to standard BP metrics such as 24h, daytime and nighttime averages of SBP, DBP and HR **(A,C,E–G)** it showcases novel dynamic metrics such as Time in Therapeutic Range [TTR, **(B)**], SBP variability **(D)**, dynamic circadian models **(H)**, dynamic SBP night-dips **(I)** and dynamic night-dip durations and morning surge acceleration **(J)**.

To showcase the potential that continual cuffless BP monitors have to depict a new generation of BP metrics, Figure 8 illustrates a set of new dynamic metrics of BP control estimated on a male patient (51 years old) during 5 months of continual monitoring by means of a commercial continual cuffless BP monitor. Panel A displays all 4,729 SBP readings performed during the monitoring period, as well as the evolution of the 24 h SBP average. SBP readings within target range (<120 mmHg) are shown as green dots, and outside of target range (≥120 mmHg) as red dots. Based on these data, panel B showcases a novel SBP-related metric of BP control: the SBP time in target range (quartiles on Y-axis: 75–100% of time = green, 50–75% of time = yellow, 25–50% of time = orange, and 0–25% of time = red), with the total percent of time spent in each quartile for monitored period on the right side. Panel C further displays the nocturnal average of SBP, with SBP >120 mmHg colored in red, and SBP <120 mmHg colored in green. Panel D displays the mid-term BP variability (SD in mmHg) for daytime (orange), nighttime (black), and average (green). Panel E and panel F display the diastolic BP average and heart rate 24-h average (green lines) and all the individual data points (green dots). Panel G displays the SBP daytime (orange), nighttime (black), and 24-h averages (green). On the same figure, shaded time periods *a, b* and *c* correspond to panel H and are also highlighted in panels I and J. Panel H showcases thus the SBP circadian patterns at time periods *a, b*, and *c* demonstrating for this patient differing patterns of night-dip, night-dip duration, and morning surge. Concerning sleep-related BP parameters, panel I showcases the tracking of the evolution of SBP night dip in mmHg (dark green) and night dip in % (light green). And panel J showcases the tracking of the evolution of the duration of SBP night-dip (red) and quantifies the morning surge (orange).

In the following sections, the most recent trends on the use of the showcased dynamic metrics of BP control are discussed, in the context of its introduction into clinical practice supported by the deployment of continual cuffless BP monitors.

#### Time in Target Range (TTR): Assessing Control Beyond Naive Averages of BP

BP is a continuous hemodynamic variable. Yet guidelines recommend treatment targets based on episodic readings. Such an assessment of “control” is not reflective of physiology for a constantly changing variable. Recently, the concept of “Time in Target Range” (TTR) has been proposed as a more practical and clinically relevant method of assessing and targeting BP control (41–43). The idea of a range of target BP range, rather than an absolute number, shifts hypertension treatment targets and strategies, and will likely advance treatment strategies and research significantly. But there is one major hurdle to implementation. This parameter is simply not possible to assess using traditional BP monitors.

Producing the number of data points to allow for accurate calculation of TTR for BP is nearly impossible using standard BP cuffs. In a *post-hoc* analysis of SPRINT (42), Fatani et al. showed that TTR was independently associated with major cardiovascular outcomes. In the trial, BP of study participants was measured in the office, using the average of three unattended readings, first monthly and then every 3 months thereafter. Study coordinators monitored and followed patients closely. This kind of evaluation is not possible on a large scale in clinical practice. Furthermore, to calculate the estimates of TTR, the intervening systolic BP was estimated using linear interpolation, a concept drawn from anticoagulation management (44). There is an assumption made in linear interpolation that the actual data value between measured data points has a reliable and consistent behavior, smoothly transitioning from one BP measured value to the next one. While this assumption may work well for anticoagulation with coumadin (with a relatively stable and predictable pharmacology in most people and settings), the same cannot be said for BP which varies widely and frequently. By their nature and design, cuffless BP monitors resolve these hurdles by providing many more data points (up to 400 average readings/month with some continual devices) in the patient's real-world environment (Figures 8A,B). Continual cuffless BP monitors are a well-suited tool to measure TTR practically, easily and at a large scale, collecting numerous daily measurements on a long-term basis.

#### BP Variability: Deploying old Physiological Hypothesis Into Large Cohorts

The association between BP variability (BPV) and increased cardiovascular risk is complex, and robust analyses have been limited by methodologic and statistical challenges. Short-term BPV has generally been assessed by ambulatory monitoring, and longer-term BPV by repeated office measures. Devices using optical sensors that provide frequent and non-disruptive BP readings around the clock will allow the first characterization of BPV in large populations. By capturing continual BPs in large populations through the day and night, we can calculate variability of daytime and nocturnal pressures (Figure 8D). Further analyses will help us identify factors associated with high or low variability of BP, thus helping elucidate cardiovascular risk profiles in the general population.

#### Nocturnal Hypertension: Extending Existing Night Metrics to Long-Term Tracking Periods

The advent of 24 h-ABPM demonstrated that the phenotype of BP is more complex than just binary variable (hypertensive or not), and it enabled the demonstration of circadian variations of BP including daytime and nighttime BP components and nocturnal physiological dipping of BP. The predictive value of these different components was compared, and each variable demonstrated predictive value regardless of absolute BP. The average nighttime BP whether systolic or diastolic was a stronger predictor of cardiovascular events (45, 46). The superior predictive value of average nocturnal BP over 24-h ABPM, daytime average BP and HBPM has been shown in both hypertensive cohorts and the general population (45, 46). This strong predictive value is remarkable since the low reproducibility of the dipping pattern has been shown in small studies and more recently in a larger study (47, 48). Indeed, only a small fraction of hypertensive patients maintain their initial dipping phenotype over 4 years (49). In addition, the tolerability of ABPM at night is less than during daytime and may hence affect sleep quality (50, 51). The sleep disturbance induced by cuff inflation has also been shown to affect the association of nighttime ABPM with outcomes (52).

Continual cuffless devices have the potential to overcome these negative features of nighttime ABPM. First, nighttime blood measurement can be repeated over days to months, allowing derivation of a more consistent nighttime phenotype. Second, without any cuff inflation the effect on sleep quality is expected to be insignificant. Nevertheless, these unique characteristics may affect the reference level of normal nighttime cuffless BP, which may need to be newly defined.

#### Phenotype-Driven Risk Assessment and Individualized Treatment: The Next Step in Hypertension Management

Beyond the metrics of BP control that have already been exposed, and by extending the existing metrics and characterizations of BP phenotypes, the deployment of continual cuffless BP monitors will trigger the generation of innovative indexes, metrics and phenotypes. All together, these phenotypes will contribute to the emergence of a new era in the diagnosis and management of hypertension, where the assessment of cardiovascular risk and the individualization/personalization of treatments and interventions will not be based on static thresholds SBP and DBP defined by population-broad guidelines, but on dynamic phenotype-driven characterization of the patients.

To stimulate the exploration of new phenotype-driven assessments of cardiovascular risk, Table 5 provides a list of existing and promising BP phenotypes that can be powered by the large scale deployment of continual cuffless BP monitors.

**Table 5:** Suggested list of BP phenotypes that can already be identified via HBPM/ABPM screening, and extended list of BP phenotypes that will be powered by the deployment of continual cuffless BP monitors.

**Legacy BP phenotypes that** **can already be identified via** **HBPM/ABPM screening**	• Sustained normotension • White coat hypertension • Masked hypertension • Sustained hypertension • Hypotension • Non-dipping / Dipping / Extreme dipping • Short term BP variability
**Innovative BP phenotypes** **that will be powered by the** **deployment of continual** **cuffless BP monitors**	• Dynamics of all previous phenotypes over weeks and months • Working days BP • Weekend days BP • Holiday BP • Dynamic BP response to intervention • Seasonal BP variability • Pregnancy BP variability • Long-term BP variability

The utility and predictive value of the classic and new phenotypes will have to be demonstrated in longitudinal epidemiological studies before we can ascertain their use in daily practice. Their independent predictive value over average 24-h or nocturnal BP will be necessary if evidence-based individual treatment is to become a reality. Studying these phenotypes will take time but could certainly provide the physician with a new panel of physiological or provoked BP responses, which may help tailoring the antihypertensive treatment in the future.

## How Continual Cuffless BP Devices Can Overcome Barriers in Clinical Management of HTN

### Solutions to Common Clinical Gaps in Treating HTN

The current structure of care delivery is poorly equipped to engage patients in their chronic disease care. Primary care providers care for about 85% of hypertensive patients and in a usual primary care visit in the US, which lasts for 15 min, an average of six medical topics are covered, with the dominant problem getting 5 min of coverage, and the remainder about 1 min each (53).

Health-focused mobile applications and wearable devices/monitors can engage patients in a much more continuous way, as the technology platforms on which they exist are commonplace and integrated into the daily lives of patients. Furthermore, technology now enables the seamless exchange of data and information between patients and providers in real-time and empowers medical decision-making. In effect, innovations extend the ability of healthcare providers to impact patients' health outside the bounds of the traditional office setting. Digital health technologies are increasingly prevalent with growing acceptance and have been shown to improve the access to quality medical knowledge and care in the underserved. All these points are consistent with the Institute of Medicine's emphasis on design, testing, and implementation of digital health solution (6, 54, 55).

Mobile devices are now ubiquitous, with 97% of Americans owning at least one cellular device; 85% of Americans, over 70% of Europeans, and 66% of Chinese own a smartphone. Furthermore, for the lower-income and ethnic minorities who shoulder a higher burden of hypertension along with less access to care—who stand to benefit the most from digital health innovations—the mobile device can be their only source of connection to the internet (smartphone dependent) (56).

**Figure 9:**
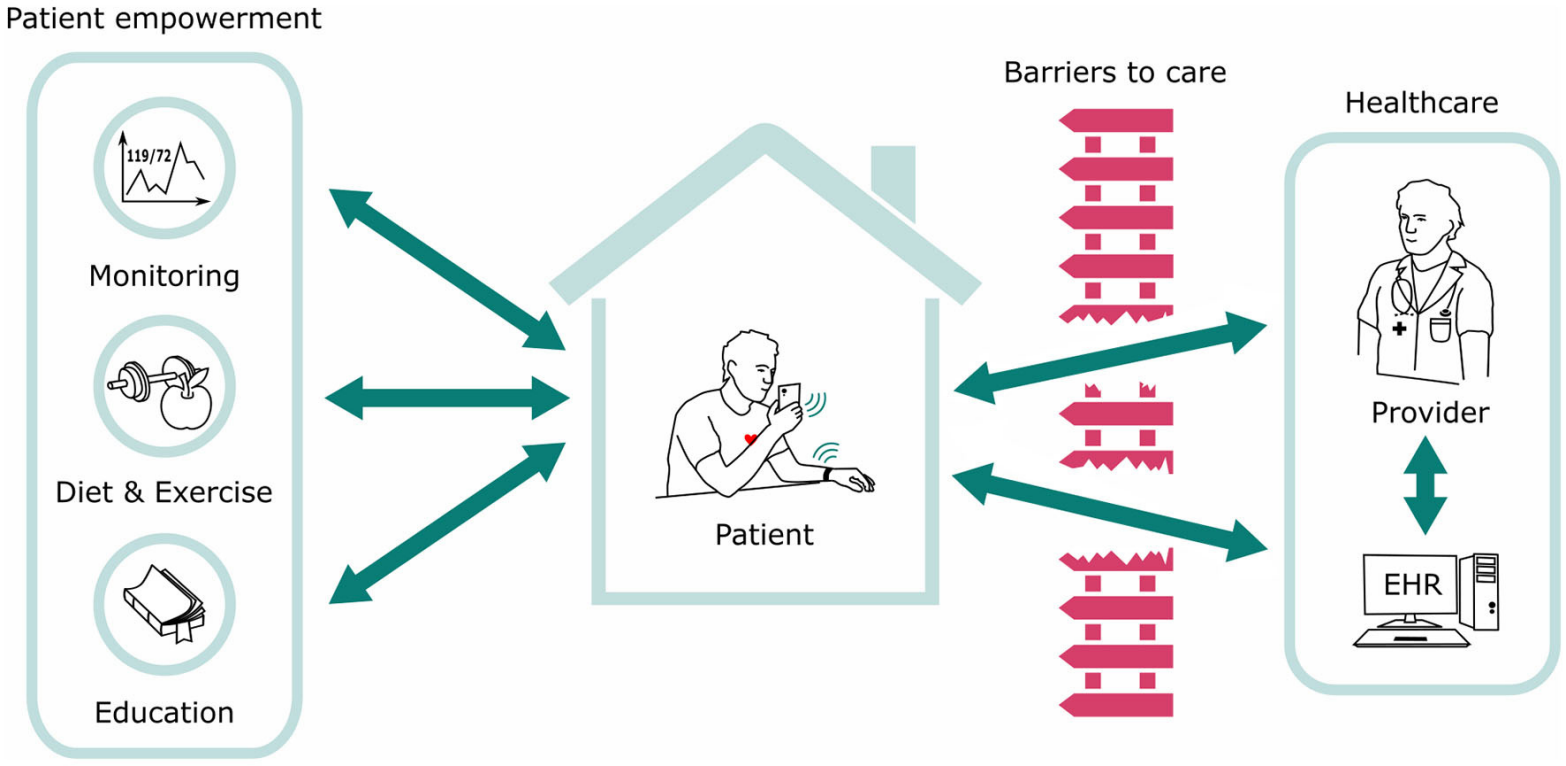
The ecosystem of Digital Health Technology and the flow of information. Cuffless BP solutions are the natural next technological step in the evolution of the ecosystem.

Innovations in healthcare technology are playing an increasingly accepted role in engaging, enabling, and empowering patients in their chronic conditions (57, 58). Concerns of data security and privacy are always prevalent but can be mitigated by careful IT processes. There are numerous applications of digital or “mHealth” to improve engagement and treatment targets in hypertension along with numerous other chronic conditions. Using digital tools to increase patient engagement and interaction with their BP readings has been associated with achieving lower BPs (59). And recently, a large study demonstrated that increasing patient engagement alone using mobile technology may be a successful tool to improve HTN in a large cohort of patients (60). Cuffless BP solutions are the natural next technological step in the evolution of digital health technology that incorporates patient engagement, enables flow of data to providers, and utilizes ubiquitous technology platforms to reach the broadest spectrum of hypertensive patients, including the underserved and highest risk patients (Figure 9).

### Therapeutic Inertia

Therapeutic inertia is a specific major contributor to difficulty in HTN management that can be overcome with continual cuffless BP monitors. The term refers to a failure of a provider to act upon or modify a treatment strategy even when targets or goals are unmet. For patients who have uncontrolled HTN documented at the time of the visit, a failure to change treatment occurs in up to 87% of encounters, for many underlying reasons (Table 6) (61–63).

**Table 6:** Reasons underlying therapeutic inertia in the management of HTN.

**Clinician**	**Patient**	**Health system**
• Underestimation of patient need • Insufficient time in the office visit • Reactive, rather than proactive decision-making	• Denial of disease/denial of disease severity • Absence of disease symptoms • Poor/lack of communication with physician • Low health literacy	• Lack of care coordination • No visit planning • Lack of decision support • No disease registry • No active outreach

In addition to these factors, the very nature of the episodic and reactive system by which most of healthcare is still governed impedes progress in chronic disease management. Continual cuffless BP solutions enable providers to monitor their patients' BP in real-time and longitudinally to show the need for up and down titration of medications, provide insights to proactively manage BP rather than waiting until the next office visit, dispel patients' denial of disease, and provide a tool for healthcare systems and payors to manage population risk more actively.

### There Will Be a Natural Transition Period Using Both Modalities

The healthcare industry has been experiencing a rapid rise in digital tools which use mobile devices with a health-related purpose. Among the numerous possible advantages of such devices are improved efficiency, empowering, engaging, educating patients, enhancing communication between patients and providers, and improved quality of life (64–67). Despite the many potential benefits of mobile health tools and devices, as well as the ever-expanding number of apps and wearable devices, adoption of routine use in clinical practice remains limited and slow. It is clear to most practicing physicians, and reinforced by studies, that digital solutions must go beyond the technology itself to drive widespread clinical adoption (68–71). The complexity, regulation, and workloads create a challenging environment in which a provider must try to adopt a new workflow. Widespread adoption of cuffless BP devices therefore must pair clinically useful technology with tailored integration into a multitude of different practice settings and systems. It must also address providers' concerns of usefulness in clinical decision-making, facilitating access to care, improving efficiency, benefitting clinical habits, ensuring privacy, all with appropriate information technology support and acceptable cost. Beyond these barriers, incorporation into expert professional society guidelines will also require studies demonstrating superior clinical outcomes using cuffless BP monitors. Only with several clinical outcomes and cost-effectiveness studies will novel technology be incorporated into standard practice.

**Figure 10:**
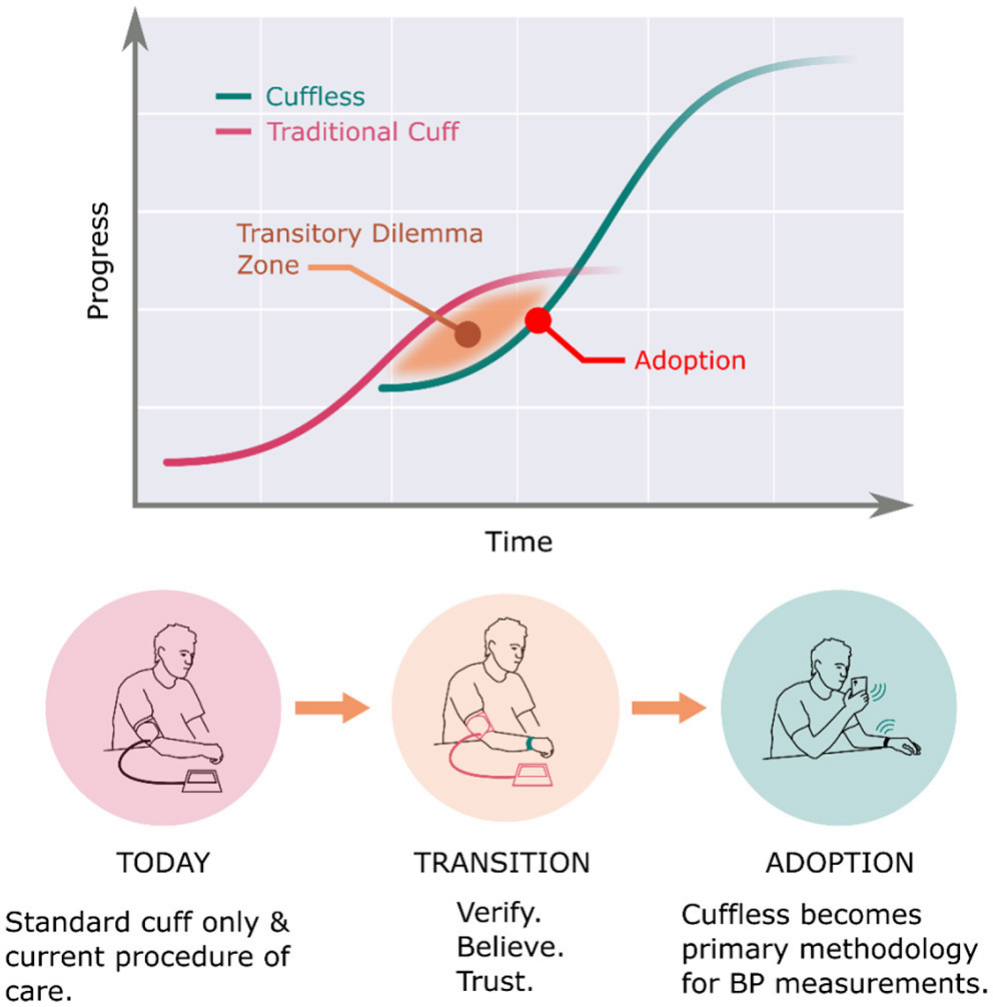
Natural transition period from standard cuff BPM to cuffless BPM.

As shown in Figure 10, BP monitors are currently in the “transition” phase from the legacy cuff monitors to newer cuffless ones. Until validation of and clinical outcomes with cuffless BP devices are established, current methods and guidelines of BP monitoring will still apply to clinical practice, even as physician innovators test and study newer devices, define optimal workflows, gain trust with the new technology, eventually incorporating cuffless technologies into guidelines and a new standard of care.

## Conclusion

For over a century the assessment of BP, a continuously changing physiologic parameter, has been founded upon a technique which inherently is unable to capture the dynamic nature of BP in daily activities and over the long term. Behavioral factors and the need for patients to play an active role in recording their BP compound these limitations. Guidelines, research, and clinical management of HTN have therefore been hampered by the capability of the primary tool of measurement, the sphygmomanometer.

Development of cuffless BP technology has now progressed far enough to begin incorporating it into research and, after validation, guidelines and clinical management of HTN. Continual cuffless BP monitoring affords patients and providers alike an opportunity to provide tremendously more BP data in a convenient, comfortable, and continual fashion. Definition, categorization, and the basic underlying principles of cuffless devices have been reviewed. The difference between the capacity of cuffless and ABPM/HBPM to generate BP data has been clearly shown, as well as suggested guidance on the interpretation of cuffless data with the framework of established techniques. Exciting novel metrics of BP such as time in target range (TTR), BP variability (BPV), nocturnal HTN, complex BP phenotyping, risk assessment, and individualized HTN management are now possible to explore, research, and implement in clinical practice.

Hypertension care needs to improve globally, and continual cuffless BP monitors can overcome many of the existing impediments to better BP management. Healthcare adoption at scale will require not only clinical validation but also tailored integration into existing workflows and EHR systems, made possible only by collaborating with healthcare providers. While it is normal to expect a transition phase of any new technology, widespread incorporation in clinical practice usually begins with leading researchers and expert associations that write guidelines. Clinical outcomes research with these devices is crucial to define, for clinical use, the optimal BP pattern and metrics with continual cuffless devices. Equipped with new such knowledge and context, it is imperative that cuffless BP monitors and data are also incorporated into future writings of society guidelines and standards.

## Data Availability Statement

The original contributions presented in the study are included in the article/supplementary material, further inquiries can be directed to the corresponding author.

## Ethics Statement

The clinical trial involving human participants was reviewed and approved by Swissmedic and Commission cantonale d'éthique de la recherche sur l'être humain (CER-VD). The patients/participants provided their written informed consent to participate in this clinical trial.

## Author Contributions

JSo, MC, DP, BD, CP, and JSh contributed to conception and design of the study. JSo, MC, and DP performed the statistical analysis. JSo and JSh wrote the first draft of the manuscript. JSo, CP, GW, NF, and JSh wrote sections of the manuscript. All authors contributed to manuscript revision, read, and approved the submitted version.

## Conflict of Interest

ML, GW, and NF consult for Aktiia. ML also is a minority shareholder of Aktiia. CP participates in clinical studies with Aktiia. The remaining authors declare that the research was conducted in the absence of any commercial or financial relationships that could be construed as a potential conflict of interest.

## Publisher's Note

All claims expressed in this article are solely those of the authors and do not necessarily represent those of their affiliated organizations, or those of the publisher, the editors and the reviewers. Any product that may be evaluated in this article, or claim that may be made by its manufacturer, is not guaranteed or endorsed by the publisher.
